# Behavioral and physiological fatigue-related factors influencing timing and force control learning in pianists

**DOI:** 10.1038/s41598-023-49226-7

**Published:** 2023-12-08

**Authors:** Mitsuaki Takemi, Mai Akahoshi, Junichi Ushiba, Shinichi Furuya

**Affiliations:** 1 Graduate School of Science and Technology, Keio University, 3-14-1 Hiyoshi, Kohoku-ku, Yokohama, Kanagawa 223-8522 Japan; 2 Sony Computer Science Laboratories, Inc., Tokyo, Japan; 3 Department of Biosciences and Informatics, Faculty of Science and Technology, Keio University, 3-14-1 Hiyoshi, Kohoku-ku, Yokohama, Kanagawa 223-8522 Japan

**Keywords:** Motor control, Biomarkers

## Abstract

Optimizing the training regimen depending on neuromuscular fatigue is crucial for the well-being of professionals intensively practicing motor skills, such as athletes and musicians, as persistent fatigue can hinder learning and cause neuromuscular injuries. However, accurate assessment of fatigue is challenging because of the dissociation between subjective perception and its impact on motor and cognitive performance. To address this issue, we investigated the interplay between fatigue and learning development in 28 pianists during three hours of auditory-motor training, dividing them into two groups subjected to different resting conditions. Changes in behavior and muscle activity during training were measured to identify potential indicators capable of detecting fatigue before subjective awareness. Our results indicate that motor learning and fatigue development are independent of resting frequency and timing. Learning indices, such as reduction in force and timing errors throughout training, did not differ between the groups. No discernible distinctions emerged in fatigue-related behavioral and physiological indicators between the groups. Regression analysis revealed that several fatigue-related indicators, such as tapping speed variability and electromyogram amplitude per unit force, could explain the learning of timing and force control. Our findings suggest the absence of a universal resting schedule for optimizing auditory-motor learning.

## Introduction

Acquiring advanced motor skills requires long-term and repetitive training, often accompanied by fatigue in the neuromuscular system. This fatigue poses the risk of developing severe injuries for those required to exert extraordinary performance, such as athletes and musicians. Thus, circumventing fatigue is crucial for achieving sustainable wellness throughout professional life. However, awareness of fatigue during training is often challenging. Previous studies have demonstrated a dissociation between the subjective perception of fatigue and its actual influence on motor and cognitive performance^1^. This discrepancy emphasizes the importance of understanding when fatigue-related physiological changes occur, which does not necessarily coincide with the moment at which fatigue is subjectively perceived^2,3^. Hence, it is necessary to identify fatigue-related markers to adjust the amount of practice based on objectively observed changes in performance or body states rather than unreliable subjective perceptions.

Potential fatigue-related indicators emerge at various levels within the neuromuscular system involved in force production and movement. These include the production of motor commands from the motor cortex through the corticospinal system, activation of motor neurons in the spinal cord, and activation of muscle fibers generating action potentials along the sarcolemma; muscle fatigue can occur at any of these stages^1,4^. Physiological changes at these stages are measurable in electromyograms (EMG), including increased amplitudes and decreased spectral frequencies while generating the same force^5,6^. The decline in maximal force, which is commonly used to define fatigue, is a typical expression of motor performance deterioration resulting from these muscular changes^7^.

The effects of muscle fatigue on motor performance depend on the characteristics of the fatiguing exercise, the task performed with the fatigued muscle, and the physiological characteristics of individual muscles. For instance, different patterns of change in response to increasing physical demands have been observed between intrinsic and extrinsic muscles^8^. When fatigue develops during a submaximal task, it can lead to the compensatory recruitment of additional motor units to counteract the decrease in force generated by the initially engaged motor units^9^. Altered patterns of muscle recruitment can compensate for fatigue in particular muscle groups so that movement output can be maintained. Furthermore, their ability to compensate for fatigue while performing complex tasks depends on their expertise^10^.

Most findings on muscular fatigue are limited to those caused by simple static tasks such as isometric force production, and little is known about the fatigue caused by complex dynamic tasks that involve coordinated movements across multiple joints and muscles. These tasks require precise control of the magnitude and timing of the exerted force, and involve controlling multiple degrees of freedom in the musculoskeletal system while receiving sensory feedback. Fatigue in such tasks is likely to be accompanied by altered patterns of functional coordination across muscles to compensate for fatigue. Consequently, performance degradation in complex tasks should be defined not only by a decline in maximal force but also by increased variability in the amplitude and timing of the force output. One representative example of a complex task suitable for addressing this issue is piano training, in which the fine control of factors such as force, speed, and spatiotemporal accuracy is crucial for generating the desired sound^11,12^. Several studies have reported that fatigue in piano training should be classified differently from fatigue in sports training, which requires high-force exertion^13^.

Additionally, the relationship between fatigue and motor learning remains unclear^10^, because it is difficult to distinguish the effects of fatigue on learning and performance^14^. Recently, one seminal study successfully disentangled the effects of fatigue from its impact on learning^15^. In this study, participants who performed a fatiguing task prior to the first day of training failed to reach the same skill level as the control group (non-fatigued on the first day), even after subsequent learning sessions on the next day under non-fatigued conditions. A recent study also suggested the involvement of the cerebellum in both fatigue perception and a decline in motor performance^16^. Nevertheless, a more detailed understanding of the relationship between fatigue and motor learning is lacking, including which muscles play an important role in motor learning and performance maintenance, and which aspects of motor skill learning are affected by fatigue.

This study aimed to address the effect of fatigue on the learning of a complex task that requires both motor and cognitive loads. We adapted an auditory-motor learning task imitating the learning of a piano sequence that requires precise force and timing control of the two fingers. We hypothesized that increasing the number of intermissions during training reduces fatigue; second, that the effects of muscle fatigue on performance differ between intrinsic and extrinsic finger muscles; and third, that subjective perception of fatigue and fatigue appearing in performance do not correlate; that is, physiological fatigue cannot be felt. To test these hypotheses, we conducted experiments that lasted for a total of three hours with two different protocols with 28 pianists.

## Methods

### Participants

Twenty-eight pianists [currently belonging to or having graduated from a college of music, including 21 females, aged 23.1 ± 3.9 years; data values expressed as means ± standard deviation (SD)] participated in the experiment. Of these 28 pianists, 14 (11 female, aged 22.9 ± 2.6 years) participated in the 1-rest condition and the remaining 14 (10 females, aged 23.3 ± 5.0 years) participated in the 2-rest condition (see “Experimental procedure” section for details). The study was conducted in accordance with the Declaration of Helsinki. The experimental procedures were approved by the Sony Bioethics Committee (approval number: 20-14-0001) and written informed consent was obtained from all participants prior to the experiments.

### Apparatus

Participants were seated in front of a table. Two force sensors (USL06-H5, Tec Gihan Co., Ltd., Kyoto, Japan) were fixed to a casing that imitated a piano keyboard, and the casing was set on the table. The participants were instructed to place their left arm on the armrest and position their middle and ring fingers on the force sensors. EMG signals were measured from the left third dorsal interosseous muscle (3DI), fourth dorsal interosseous muscle (4DI), extensor digitorum communis (EDC), and flexor digitorum superficialis (FDS) muscles using wireless surface EMG sensors (Trigno Quattro/Mini, Delsys, Natick, MT, USA). Two electrodes on the EMG sensors maintained a fixed distance; one above the muscle belly and the other distally. Skin was prepped with alcohol to reduce the impedance. A computer monitor placed in front of each participant displayed the task content.

Force sensor signals were sent to a computer (G-Tune E5-165, MouseComputer Co., Ltd., Tokyo, Japan) through an analog-to-digital converter (USB-6363, National Instruments, Austin, TX, USA) at a sampling rate of 1000 Hz. The computer calculated the sound volume based on the magnitude of the finger force using the following equation:

$$V\left( t \right) = 75\frac{{{\text{ln}}\left( {1 + F\left( t \right) - \beta } \right)}}{{{\text{ln}}\left( {1 + \alpha - \beta } \right)}}$$

where *V*(*t*) represents the percentage of the sound volume playing from the computer at time *t*, relative to the maximum volume, and *F*(*t*) is the average finger force over the last 20 ms. The constant α corresponds to a force level equal to one-third of the participant’s maximum voluntary contraction (MVC) (see “MVC test” section for details on MVC calculation), and β represents the baseline level that was determined by the mean + 2SD in the period when participants placed a finger on the force sensor for a few seconds without intentional force application. Following a previous study, we converted the log of the finger force to sound^17^. The MVC and baseline levels were measured before the start of the training session. A 400 Hz sound (close to the G sound) was generated when the force sensor placed under the middle finger was pressed. Pressing the sensor under the ring finger generated a 300 Hz sound (close to the D sound).

EMG signals were first sent to the base station, where the data were band-pass filtered (20–450 Hz, Butterworth filter), and then sent to the computer, which also received analog data from the force sensors through an analog-to-digital converter. Both the force and EMG data were stored on a computer for offline analysis. The experimental program was coded using LabView 2021 software (National Instruments).

### Experimental procedure

**Figure 1:**
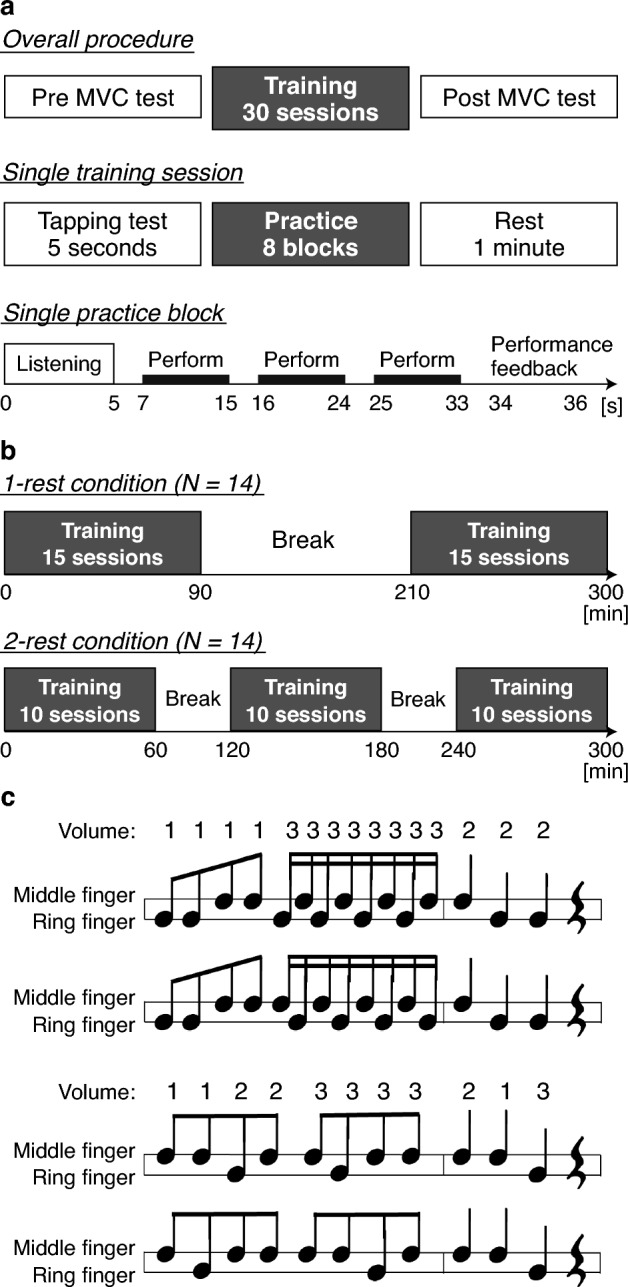
(**a**) Experimental procedure. Thirty training sessions were conducted before and after MVC tests. A single training session consisted of a tapping test followed by eight practice blocks. Subjective rating of perceived exertion was obtained after the completion of the eight practice blocks. Participants were instructed to perform the same sequence they listened to three times within each practice block. One of the four sequences shown in (**c**) was randomly played in each practice block, with each sequence being played twice within a single training session. (**b**) Experimental conditions. (**c**) Task sequences. The sequences were played at a tempo of 96 beats per minute.

The experiment comprised 30 training sessions. Before and after the 30 training sessions, the MVC of the participants’ middle and ring fingers were assessed independently (Fig. 1a, top). While the experimenter counted seconds, participants increased their finger force in the first 2 s until it reached the maximum they could exert and maintained the maximal force for 2 s afterward. The measurements were repeated three times, and the measurement with the highest force was used for analysis. MVC was also measured before and after each break; however, these measurements were not used in the present study.

A single training session consisted of eight blocks (Fig. 1a, middle). In one block, the participants listened to the model’s performance (listening trial) and tried to imitate it three times as precisely as possible (performing trial) (Fig. 1a, bottom). The music sheet of the sequence was shown on a computer monitor in front of the participant from the start of the model performance until the last trial. Because the music sheet did not indicate the sound volume, the participants had to remember the volume changes from the listening trial and imitate them. Each performing trial started with a ‘GO’ cue on the computer monitor. After three trials, the average score, reflecting the accuracy of volume, rhythm, and keypress duration, appeared on the computer monitor (performance feedback). The total score was 100, of which 50 was allotted to volume accuracy, 25 to rhythm accuracy, and 25 to keypress duration accuracy. Model performance was randomly chosen from the four sequences (Fig. 1c).

The participants were grouped into two different training conditions. In the 1-rest condition, the experiment was divided into two sets of 15 training sessions with one 120-min rest, and the 2-rest condition was divided into three 10 training sessions with two 60-min rests (Fig. 1b). Between training sessions, participants took a 1-min rest during which they performed a 5-s tapping task and completed a fatigue questionnaire. During the tapping task, the participants alternately tapped their middle and ring fingers as quickly as possible. The CR-10 Borg scale was used as the fatigue questionnaire^18^.

### Data analysis

#### Behavioral data during training session

The raw force data measured by the force sensors were smoothed over 20 ms and segmented into single trials. Key presses were detected by identifying periods where the force level exceeded the baseline level. The peak force and timing of each keypress were then determined. The success rate was calculated as the number of trials in the correct sequence order divided by the total number of sequences performed by the participant (24 trials/session). This was then converted into percentages. The timing error was calculated as the absolute difference between the detected peak timing and the sample sequence. The force error was calculated as the absolute difference between the detected and expected peak forces to generate the sound volume of the sample sequence, which was normalized by the participant’s MVC and converted into a percentage. Finally, the timing and force error values were individually averaged for each training session using data from the trials in which the task was successfully completed.

To quantify the time to best performance and the amount of learning, we performed curve fitting on the individual timing and force error data. These curve fittings were separately performed for the results of sessions 1–15 and 16–30 in the 1-rest condition and sessions 1–10, 11–20, and 21–30 in the 2-rest condition. We expected two types of error change trends during the training period: exponential decay, indicating consistent motor learning, and a decrease followed by an increase, reflecting both learning- and fatigue-induced deterioration in performance. Hence, we applied both the exponential and quadratic functions and compared the goodness of fit using the adjusted R^2^. The specific functions used for curve fitting are as follows:

$$E\left( n \right) = A_{e} \exp \left( {B_{e} n} \right) + C_{e}$$

$$E\left( n \right) = A_{q} n^{2} + B_{q} n + C_{q}$$

here E(n) represents the average error in training session *n*, where *n* is the number of training sessions since the last break (e.g., *n* = 1 for the 16th training session in the 1-rest condition, and *n* = 6 for the 2-rest condition). The parameters A, B, and C were estimated through the ‘nlinfit’ function in MATLAB 2021b (Mathworks, Natick, MA, USA).

**Figure 2:**
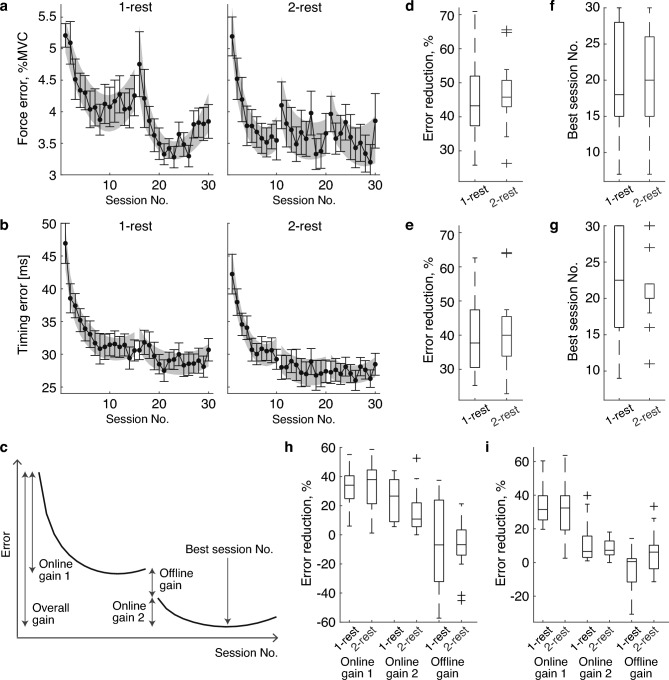
Error reduction over 30 training sessions. (**a**,**b**) Force error and timing error throughout the training sessions. The left and right figures show the results for the 1-rest and 2-rest conditions, respectively. Black dots and error bars represent the mean and standard error of the mean (SEM) of the empirical results, respectively. The gray shaded areas represent the SEM of the individual curve fitting results. (**c**) Schema of learning indexes. (**d**,**e**) Overall gain, calculated as the difference between the highest and lowest force (**d**) and timing errors (**e**) observed across the training sessions. (**f**,**g**) Best session number, indicating the session number with the lowest force (**f**) and timing error (**g**). (**h**,**i**) Online and offline gain for force and timing error, respectively.

Following curve fitting, the individual error for each session was estimated using either exponential or quadratic functions depending on the goodness of fit analysis results. Five learning-related indices were calculated to quantify the time to the best performance and the amount of learning (Fig. 2c). (i) Best session number: The session number showing the minimal error, where a smaller value indicates a shorter time to achieve the best performance. (ii) Overall gain: difference between the maximum and minimum errors over 30 training sessions. (iii) Online gain 1: the difference between the maximum and minimum errors over the training sessions before the first break. (iv) Online gain 2: the difference between the maximum and minimum errors during the training sessions after the first break. (v) Offline gain: the difference in error between the last session before the first break and the first session after the first break.

#### MVC test

Using the data from the EMG and force sensors, we calculated the MVC, EMG median frequency (EMG-MF), and EMG amplitude per unit force (EMG/force). The raw EMG signal was subjected to full-wave rectification and smoothed with a 40 ms moving average. For the MVC calculation, we averaged the force during the middle 1 s interval within 2 s in which the maximum force was maintained. As we measured the maximum force of the left middle and ring fingers separately, the MVC value was represented by the average of the MVC exerted by these two fingers. EMG-MF and EMG/force were also determined using the same two 1-s data segments. EMG-MF was computed using the ‘medfreq’ function in MATLAB 2021b. The EMG/force ratio was obtained by dividing the average preprocessed EMG amplitude by the MVC.

#### Tapping test

We computed the tapping speed and speed variability using data derived from the force sensors. The tapping speed was calculated as the reciprocal of the average tapping interval, and variability was represented by the SD of the tapping interval divided by the average interval.

#### Statistical analysis

In this study, several parameters related to force and timing error reduction were evaluated. These included the session number representing the smallest force error (best session number); the amount of overall, online, and offline error reduction; the highest perceived exertion; changes in the average maximum finger force between the pre- and post-MVC tests; differences in the mean tapping speed and mean tapping timing variability between the first five and the last five tapping tests; and changes in the EMG-MF and EMG/force of the four muscles at the pre- and post-MVC tests. To compare these variables between the 1-rest and 2-rest condition groups, we employed either the Wilcoxon rank-sum test or the two-sample t-test, depending on the data distribution.

Furthermore, we conducted group-wise comparisons of changes in force error, timing error, and task success rate. For this analysis, we employed an analysis of variance (ANOVA) with the session number (1, 2, …, 30) as the within-subjects factor and the group (1-rest and 2-rest conditions) as the between-subjects factor. Post hoc pairwise comparisons were performed using Tukey’s test to correct for multiple comparisons. The type I error was set to 0.05 for all statistical analyses. JASP 0.16.2 (Apple Silicon) was used for statistical analyses.

#### Stepwise regression analysis

To assess factors that affect different aspects of motor skill learning, we performed stepwise regression analyses using the default setting of the ‘stepwiseglm’ function in MATLAB 2021b. We set the parameters related to force and timing error reduction (i.e., the best session number and the amount of overall, online, and offline error reduction) as the response variables. Predictor variables included differential values of MVC of the middle and ring fingers, EMG-MF and EMG/force of 3DI, 4DI, EDC, and FDS muscles, maximal tapping speed, tapping timing variability, and maximal perceived exertion score before and after the training sessions. Both the dependent and independent variables were standardized prior to the regression analysis.

## Results

### Effect of rest frequency and timing on force and timing error reduction

Figure 2a,b illustrate the group averages of force and timing errors for each session in the 1-rest and 2-rest conditions, respectively. Two-way ANOVAs revealed no interaction between session and group for force error (F(29, 754) = 1.39; *p* = 0.087) or timing error (F(29, 754) = 0.70; *p* = 0.88). No main group effects were observed for force (F(1, 26) = 0.82, *p* = 0.37) or timing errors (F(1, 26) = 0.88, *p* = 0.36). These findings suggest that the changes in force and timing errors do not depend on the number of rests. However, both force and timing errors exhibited significant main effects of the session (F(29, 754) = 6.22 and 24.8, respectively; *p* < 0.001 for both), indicating that the participants successfully learned motor skills.

To remove the variation among sessions, we quantified the time to best performance and the amount of learning through curve fittings of the individual timing and force error data, as shown in Fig. 2d–g. The Wilcoxon rank-sum test found no significant differences in the time to best performance or the amount of learning between the two conditions (*p* > 0.05 for all comparisons). Furthermore, no group differences were found for online and offline gains, as shown in Fig. 2h–i. The detailed statistical analysis results are presented in Table 1.

**Table 1:** Statistical analysis results.

Figure no	Test method	Dependent variable	Statistic
2d	Wilcoxon rank-sum test	Force, overall gain	− 0.391
2e	Wilcoxon rank-sum test	Force, best session no	0.000
2f	Wilcoxon rank-sum test	Timing, overall gain	− 0.115
2g	Wilcoxon rank-sum test	Timing, best session no	0.533
2h	Wilcoxon rank-sum test	Force, online gain 1	− 0.391
2h	Wilcoxon rank-sum test	Force, online gain 2	1.815
2h	Wilcoxon rank-sum test	Force, offline gain	0.437
2i	Wilcoxon rank-sum test	Timing, online gain 1	0.574
2i	Wilcoxon rank-sum test	Timing, online gain 2	0.207
2i	Wilcoxon rank-sum test	Timing, offline gain	− 1.585
3a	Wilcoxon rank-sum test	Max. perceived exertion	− 0.379
3b	Wilcoxon rank-sum test	ΔMVC	0.161
3c	Wilcoxon rank-sum test	ΔTapping speed	− 0.718
3d	Two-sample t-test	ΔTapping timing variability	1.552
3e	Wilcoxon rank-sum test	ΔEMG/force, EDC muscle	0.170
3f	Wilcoxon rank-sum test	ΔEMG/force, FDS muscle	0.849
3g	Two-sample t-test	ΔEMG/force, 3DI muscle	0.316
3h	Two-sample t-test	ΔEMG/force, 4DI muscle	0.269
3i	Wilcoxon rank-sum test	ΔMF, EDC muscle	− 0.267
3j	Wilcoxon rank-sum test	ΔMF, FDS muscle	− 2.208
3k	Wilcoxon rank-sum test	ΔMF, 3DI muscle	− 1.179
3l	Wilcoxon rank-sum test	ΔMF, 4DI muscle	− 0.540

While not statistically significant, the speed of force error reduction appeared faster in the 2-rest condition, particularly in the early learning phases (e.g., the first ten training sessions). This might be due to between-group differences in task success rate during the early learning phase (Supplementary Fig. 1). In fact, a two-way ANOVA revealed a significant interaction between session and group for the success rate (F(29, 754) = 1.67, *p* = 0.013), and a post hoc test showed that the success rate in the first training session was significantly higher for the 2-rest condition than for the 1-rest condition (*p* < 0.05). Participants in the 1-rest condition may have been too engaged to accurately follow the assigned sequences, leading to challenges in precise force control.

### Impact of rest conditions on fatigue indicators

**Figure 3:**
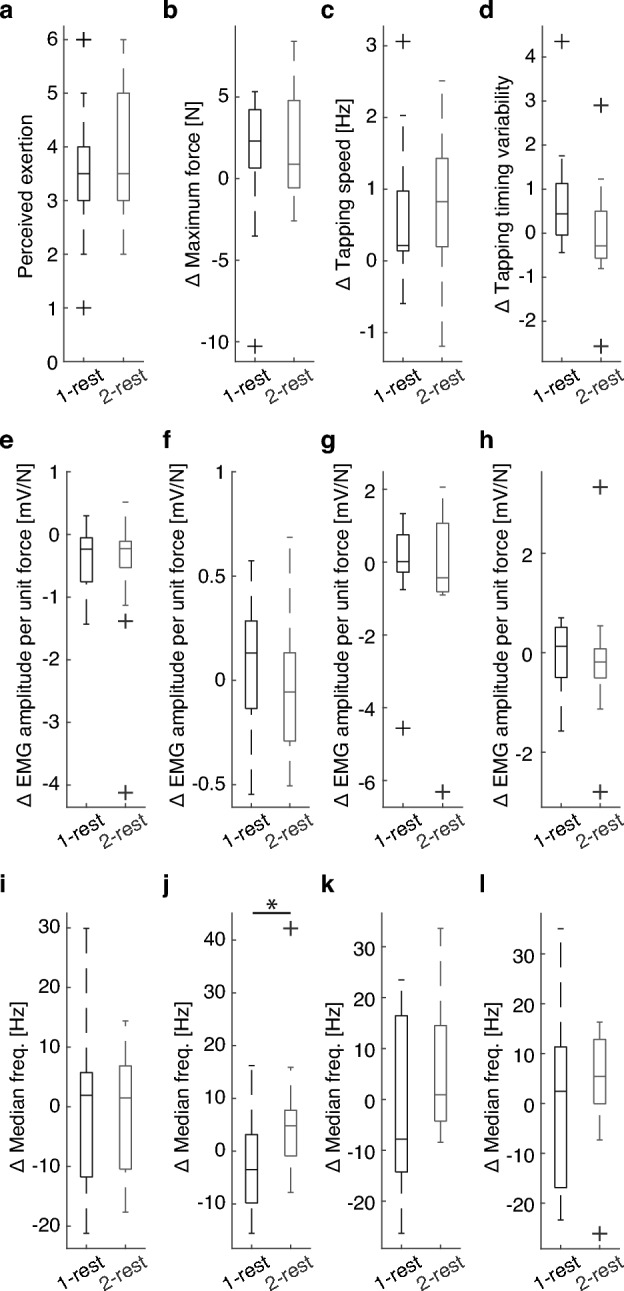
Changes in fatigue indicators over the training sessions. (**a**) Highest perceived exertion across 30 training sessions. (**b**) Difference in the average maximum force of the left middle and ring fingers between the pre- and post-MVC tests. (**c**) Difference between the mean tapping speed and (**d**) the mean timing variability (i.e., coefficient of variation) for the first five tapping tests and the last five tapping tests. (**e–h**) Changes in EMG median frequency of FDS, EDC, 3DI, and 4DI muscles at the pre- and post-MVC tests, respectively. (**i**–**l**) Changes in the ratio of EMG amplitude of FDS, EDC, 3DI, and 4DI muscles to the average maximum force at pre and post MVC tests, respectively. *p < 0.05 by a non-parametric pairwise comparison.

We quantified the fatigue indicators using data from the MVC and tapping tests. Pairwise comparisons revealed no significant differences in the fatigue-related metrics between the groups, except for the EMG-MF of the FDS muscle. The highest perceived exertion across the 30 training sessions showed no significant difference between the two groups (Fig. 3a; *p* = 0.705). The change in the average maximum force of the left middle and ring fingers between the pre- and post-MVC tests was also similar between the groups (Fig. 3b; *p* = 0.87). Similarly, there were no significant differences in the mean tapping speed (Fig. 3c; *p* = 0.473) or mean tapping timing variability (Fig. 3d; *p* = 0.134) for the first and last five tapping tests. Changes in EMG/force at pre- and post-MVC tests were also not significantly different between the groups (Fig. 3e–h; *p* = 0.40–0.87). However, the Wilcoxon rank-sum test identified a significant group difference in the changes in the EMG-MF of the EDC muscle between pre- and post-MVC tests (Fig. 3j; *p* = 0.027). Note that EMG-MF did not significantly differ between the groups for the other muscles (Fig. 3i,k,l; *p* = 0.24–0.79). The detailed statistical analysis results are presented in Table 1.

### Covariation of tapping timing variability and EMG amplitude per unit force with motor skill learning

**Figure 4:**
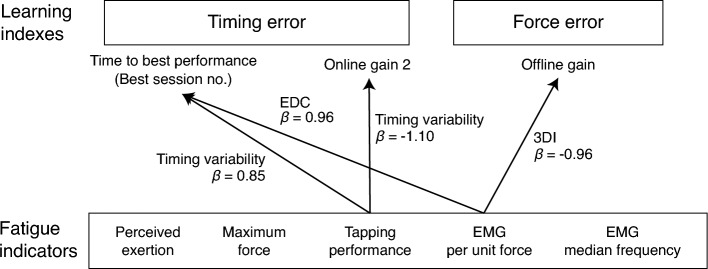
Dependencies between learning indexes and fatigue indicators. These results were derived from stepwise regression analyses.

Figure 4 shows the relationships between the learning indices (response variables) and fatigue indicators (predictor variables) derived from the stepwise regression analysis. We observed a positive association between the time to best performance in timing error and changes in the EMG/force of the EDC muscle, as well as tapping timing variability between the pre- and post-MVC tests. This implies that participants whose EMG/force and tapping timing variability increased after training required more time to acquire complex auditory-motor skills. A post-training increase in tapping timing variability was also linked to a smaller online gain 2 in timing error. Furthermore, participants whose 3DI muscle EMG/force increased after training exhibited a smaller offline gain in force error. Notably, perceived exertion was not linked to any of the learning indices. These findings suggest that the tapping timing variability and EMG/force may help predict the efficiency of learning dexterous movements. However, none of the fatigue indicators explained the amount of overall learning gain throughout the training.

## Discussion

The present study has two key findings. First, motor skill learning and fatigue evolution may be independent of resting frequency. Learning indices, such as reduction in force and timing error throughout training, were not different between the two groups with different numbers of rests. Similarly, we observed no difference in fatigue indices, such as the MVC test, tapping test, muscle activities during those tests, and perceived exertion, between the two groups. This finding suggests a counterintuitive insight: there may not be a uniform resting schedule for the enhancement of learning efficiency, although other unexplored features have the potential to reveal the effects of a resting strategy on learning. Second, several fatigue-related indicators may explain the learning of timing and force control. Regression analyses revealed that the tapping speed variability explained the time-to-best performance and online gain in timing error, whereas the EMG amplitude per unit force during the MVC test explained the time-to-best performance in timing error and offline gain in force error. Together, these results highlight the unreliability of the subjective perception of fatigue, and thus, the necessity of an objective test battery, such as the tapping test, to reliably evaluate fatigue.

Contrary to our hypothesis, frequent rest did not lead to enhanced learning. Our hypothesis was based on several neurophysiological factors such as memory consolidation during rest^19,20^, offline learning^21^, and learning deterioration under fatigue^15^. One potential reason for this counterintuitive result may be the limited understanding of the mechanisms governing recovery from muscle fatigue during rest. While endurance against fatigue has been actively investigated^22^, little is known about recovery from fatigue, such as the factors underlying prolonged central fatigue accompanying long-duration exercise and the effect of exercise intensity and duration on the time course of neuromuscular recovery^23^. Our results suggest that muscle recovery from fatigue may play a role in the efficiency of motor skill learning, although the precise relationship remains unclear.

Our results showed notable inter-subject variability in the change in fatigue indicators over the training sessions, despite all the subjects being trained pianists. This finding aligns with a number of studies that have reported subject-specific neuromuscular responses to fatigue that appear in MVC and EMG^24,25^. One putative factor that explains individual differences in the amount of fatigue among pianists is their motor skills in reducing unnecessary muscular activity during piano keystrokes^12,26,27^. For example, expert pianists can utilize more non-muscular forces, such as inertial and gravitational forces, thereby reducing the muscular load^26,27^. Pianists who exerted a shorter duration of muscular work exhibited faster piano performances, highlighting the inter-individual differences in neuromuscular skills as a key expertise of pianists^12,28^. Another factor is the endurance of the finger muscles against fatigue, which is likely superior in pianists than in non-musicians^13,29^. Physiological adaptation to muscular fatigue in pianists suggests piano training has the nature of endurance training, which can differ across pianists depending on the amount and ways of practicing.

A key finding of our study is that tapping speed variability, rather than the tapping speed itself, serves as an indicator of fatigue that can explain motor skill learning. It has been reported that the inhibitory M1-intracortical circuits and corticospinal excitability increase during unresisted repetitive movements^30^. They also observed that the MVC force and the level of the central drive to the muscle remained unchanged after 30 s of finger tapping. Another study reported that while short-lasting repetitive movements induce fatigue within intracortical inhibitory circuits, isometric contractions have a clear impact on spinal circuits^31^. These findings suggest that a task that does not require isometric contraction, but rather fine control of force and timing, triggers fatigue within the intracortical circuits and not merely at the muscle level. This could explain why tapping speed variability can be a predictor of learning but not speed itself, which might be a more direct fatigue indicator. One possible reason for the unchanged tapping speed in our experiment is the relatively short duration (5 s) of the tapping task. Future observations of the concomitant shift in the movement rate and firing rate of motor neurons, as reflected in the median frequency of EMG, toward a lower frequency with fatigue, may signify synchronization between different systems, aligning with Bernstein's classical statement^32^.

Another fatigue-related indicator that seemed to explain the detrimental effect on motor skill learning was the EMG amplitude per unit force. This result agrees with those of previous studies demonstrating that as fatigue sets in, the EMG amplitude required to exert the same force tends to increase^5,6^. The increase in motor unit activity reflected in the EMG amplitude can include changes in the number of active motor units and modulation of the discharge rate to compensate for peripheral fatigue^33^. Because the EMG per unit force is a clear indicator of fatigue, which has been reported in several previous studies, our results extend those of a previous study that demonstrated deteriorated learning under muscle fatigue^15^. However, drawing a more reliable conclusion requires performing a cross-validation of the features in the regression model in future studies, which the present study could not do because of the small sample size. Additionally, recognizing potential contributors to result variability, such as hormonal influences associated with the menstrual cycle, known to impact feelings of fatigue and finger fluency^34,35^, could further enrich our understanding.

Fatigue detection is crucial to avoid neuromuscular disorders triggered by overtraining. The present study aimed to discover potential markers to detect fatigue even when a pianist does not consciously perceive it. The results suggest the potential utility of tapping speed variability and EMG per unit force as reliable fatigue indicators in the context of auditory-motor learning. In addition, our results suggest the need to tailor rest periods for each individual during learning for trained pianists. It is worth noting that resting, which is not well designed for individuals, does not enhance learning efficiency. As the neuromuscular system of professional pianists for auditory-motor learning is optimized after years of musical training^36^, it is crucial to adopt test batteries, such as the tapping test, to correctly identify fatigue that appears differently among individuals.

Further investigation is required to verify the practical application of tapping speed variability and EMG per unit force as fatigue indicators. While the task adapted in this study was similar to piano training in that it required fine force and timing control, it was not representative of typical piano practice. This is because it involves only two fingers and utilizes an apparatus that is not a real piano. To apply these indicators in the actual piano practice of professionals, future studies must test the capacity of these markers to detect fatigue in real piano practice.

## Conclusions

We tested the effect of fatigue on the learning of a complex task, by conducting an auditory-motor learning task experiment in two groups with different resting conditions. Contrary to our hypothesis, the results showed no uniform resting schedule for optimizing auditory-motor learning. Instead, our results suggest the possibility of using additional objective tests during training, such as a tapping test, to detect fatigue and optimize individual skill learning. With further investigations to verify their aptitude as fatigue detectors, this finding can be applied to musical training and other types of motor learning, such as sports and rehabilitation.

### Supplementary Information

Supplementary Information.

## Data Availability

Data to generate all plots in Figs. 2 and 3 can be found at https://osf.io/mvhxn/. The other data (e.g., raw data) will be provided upon request to the corresponding authors.
