# Supplementary material for: Behavioral and physiological fatigue-related factors influencing timing and force control learning in pianists
Source: Sci Rep. 2023 Dec 8;13:21646. doi: 10.1038/s41598-023-49226-7 (PMC10703774; doi:10.1038/s41598-023-49226-7)
Supplement: Supplementary file 1 — Supplementary Information. [file 41598_2023_49226_MOESM1_ESM.pdf]

Behavioral and physiological fatigue-related factors influencing timing and force control learning in pianists

Authors

Mitsuaki Takemi[1#], Mai Akahoshi[2, 3#], Junichi Ushiba[3], Shinichi Furuya[2]

Affiliations

[1]Graduate School of Science and Technology, Keio University, Kanagawa, Japan;

[2]Sony Computer Science Laboratories, Inc., Tokyo, Japan;

[3]Department of Biosciences and Informatics, Faculty of Science and Technology, Keio University, Kanagawa, Japan;

[#]Equally- contributed co-first authors

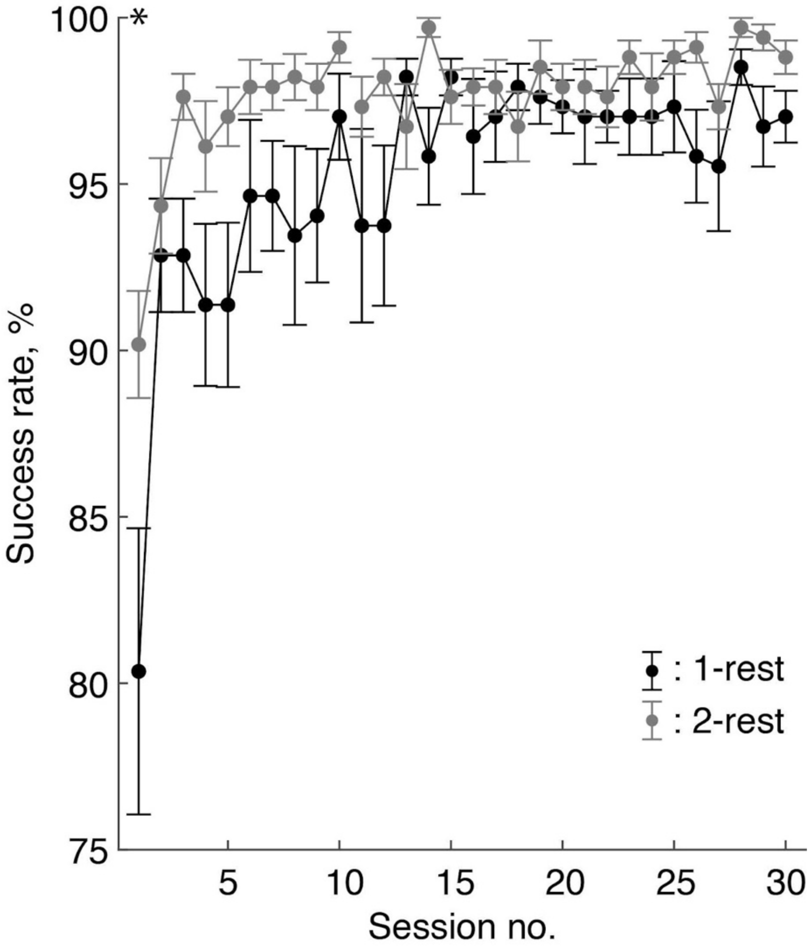

Supplementary figure 1. Task success rate across the training sessions. The filled dots and error bars represent the mean and standard error of the mean, respectively. *: $p < 0.05$ by post-hoc pairwise comparisons.
